# The roles of p38MAPK and caspase-3 in DADS-induced apoptosis in human HepG2 cells

**DOI:** 10.1186/1756-9966-29-50

**Published:** 2010-05-18

**Authors:** Chunxiao Ji, Fenglian Ren, Heng Ma, Ming Xu

**Affiliations:** 1 College of Chemistry and Chemical Engineering, Central South University, Changsha, Hunan 410083, China; 2 Research Institute for Molecular Pharmacology and Therapeutics, Central South University, Changsha, Hunan 410083, China

## Abstract

**Objectives:**

To explore the function of p38MAPK and caspase-3 in DADS-induced apoptosis in human HepG2 cells, and discuss the signal transduetion mechanism of HepG2 cells in the apoptosis process induced by DADS by using the inhibitors of p38MAPK (SB203580) and caspase-3 (Z-DEVD-FMK).

**Methods:**

After the human HepG2 cells had been treated with the DADS and inhibitors for 24 h, cell viability was determined by the MTT method, apoptosis was evaluated by flow cytometry (FCM) and the expressions of p38MAPK and caspase-3 were measured by western-blot.

**Results:**

Our results indicated that DADS activities the p38MAPK and caspase-3, but the inhibitors, SB203580 and Z-DEVD-FMK (for p38MAPKand for caspase-3, respectively), both have the effect of inhibitory activity on P38MAPK and caspase-3. Furthermore, a combination treatment with both DADS and inhibitor (SB203580 or Z-DEVD-FMK) decreases the inhibitory and apoptotic activity of HepG2 cells increased compared with DADS-treated.

**Conclusions:**

Our data indicate that p38MAPK and caspase-3 are involved in the process of DADS-induced apoptosis in human HepG2 cells and interact with each other.

## Background

The MAPK (mitogen-activated protein kinase) system is a cluster of serine/threonine protein kinases in the cells, and the activitied MAPKs participate in a variety of cellular responses including genetic transcription, inducing cell apoptosis, maintaining cell and regulating cell cycle, and so on [1-3]. The p38MAPK is the key member of the MAPK family and more commonly activated in response to cytokines, stress and cellular damage [4,5]. A large number of studies have shown that the activity of p38MAPK is necessary in the apoptosis process induced by various anti-cancer drugs. Caspase enzymes play a very important role when cells started apoptosis as the central effector of apoptosis. Caspase-3, is the ultimate enforcer of apoptotic death, which can cleavage many proteins of important structure and function directly[6].

Diallyl disulfide (DADS) is one kind of oil-soluble sulfur organic compounds, it is a potential broad-spectrum anti-cancer drug. Studies have shown that DADS can inhibit human tumor cells grow including those of colon, lung, skin, breast, liver origins and prostate [7-10]. There are also lots of reports about the caspase-3 involvement during apoptosis process with DADS induction, such as The DADS induced apoptosis by the activation of caspase-3 in human leukemia HL-60 cells in a dosedependent manner, DADS promoted caspase-3 activity and increased cyclin E and decreased CDK2 gene expression which may lead to the G2/M arrest of T24 cells, Effects of diallyl disulfide (DADS) on expression of apoptosis associated proteins in androgen independent human prostate cancer cells (PC-3) [11,12], and so on. Our previous studies have shown that the activated p38MAPK appears to play a cytoprotective role, and the MAPK specific inhibitors enhance apoptotic effects in HepG2 hepatoma cells with DADS treatment[13]. In this report we used the inhibitors of p38MAPK (SB203580) and caspase-3 (Z-DEVD-FMK) to detect the relation of p38MAPK and caspase-3 in the apoptosis process induced by DADS, we found that p38MAPK and caspase-3 are involved in the process of DADS-induced apoptosis in human HepG2 cells and interact with eachother.

## Materials and methods

### Major reagents

DADS (80% purity) was purchased from Fluka Co., Dulbecco's modified Eagle medium (DMEM) medium, BSA and SB203580 were purchased from Sigma. Z-DEVD-FMK was purchased from CALBIOCHEM (USA), goat horseradish peroxidase (HRP)-conjugated anti-rabbit secondary antibody were purchased from Santa Cruz Biotech. Antibodies to p38, phospho-p38 (p-p38), caspase-3 were purchased from Cell Signaling.

### Cell culture

HepG2 (the human hepatoma cell line) were provided by the Xiangya school of medicine and cultured in DMEM with 10% heat-inactivated fetal bovine serum (FBS), benzylpenicillin (100 kU/L) and streptomycin (100 mg/L) at 37°C in an incubator containing humidified air with 5% CO_2_.

### Cell viability assay

Cells were seeded into 96-well plates at 1 × 10^4 ^cells per well 24 h before treatment. The cultures were then rinsed in phenol-free DMEM medium and incubated with respective test substances in phenol-free and serumfree DMEM for 24 h. In the inhibition test, Cells were treated with DADS after being treated with inhibitors 30 min. At the end of this time interval, 20 μl (5 mg/ml) MTT [3-(4,5dimethylthiazol-2-yl)-2,5-diphenyltetrazolium bromide] was added to each well, and after incubation at 37°C for 4 h the MTT solution was removed and 200 μl of dimethylsulfoxide (DMSO) was added to dissolve the crystals. The absorbance of each well at 570 nm was measured.

### Flow cytometry analysis

Cells were seeded into 100 ml cell culture bottles at 12 × 10^6 ^cells 24 h before treatment. Then cells were treated according to the aforementioned method and incubated for 24 h. Afterwards, cells were collected, made into single cell suspension and centrifuged at 800 g for 5 min. Discard the supernatant, washed cells three times with the cool PBS and fixed them 24 h with cool alcohol at 4°C. Taked 1 ml cell suspension (10^6^/ml), washed it three times with the cool PBS, treated it with RNase for 30 min at 37°C, and stained it with PI for 30 min at 37°C in a dark environment. Then the flow cytometry analysis can be carried out.

### Western-blotting

Taked the cells in the logarithmic growth phase, treated them according to the aforementioned method and incubated for 24 h. After fragmentation on ice for 20 min, the lysates were centrifuged at 15,000 *g *for 10 min at 4°C, collected the protein and quantitated it with the BCA method, electrophoresed and isolated protein by the SDS-PAGE (10%), used the electrotransfer method, carried out the blocking and hybridization on the cellulose nitrate film, detected the protein expression of cells using the ECL western blotting method. The densities of protein bands were calculated using the Quantyone software.

### Statistics

Data are expressed as mean ± S.D of three independent experiments and evaluated by one-way analysis of variance (ANOVA). Significant differences were established at P < 0.05.

## Results

### Changes of cell activity

**Figure 1:**
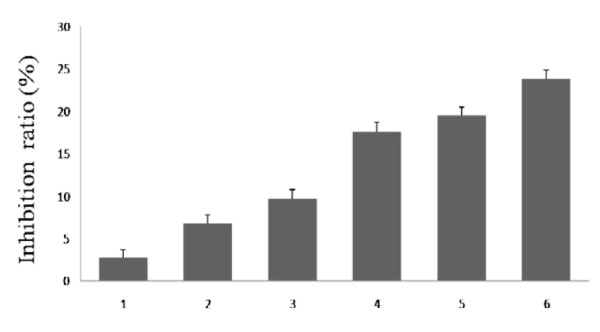
**Results of the MTT assay**. Lane 1: control (untreated), lane 2: Z-DEVD-FMK (10 μmol/L), lane 3: SB203580 (10 μmol/L), lane 4: treated with DADS (100 μmol/L) after being treated with SB203580 (10 μmol/L) for 30 min lane 5: treated with DADS (100 μmol/L) after being treated with Z-DEVD-FMK (10 μmol/L) for 30 min, lane6: DADS (100 μmol/L). Cells viability was determined by MTT assay as described in Materials and Methods. Data are expressed as mean ± S.D and evaluated by one-way analysis of variance (ANOVA). Results are representative of three replicates (P < 0.01).

Cell viability was determined by the MTT assay. As shown in Figure 1. After treatment and incubated for 24 h, the inhibition ratio of treated with 10 μmol/L SB203580 and 100 μmol/L DADS was 19.45% at 24 h, and the inhibition ratio of treated with 10 μmol/L Z-DEVD-FMK and 100 μmol/L DADS was 17.64% at 24 h, both of them were lower than the inhibition ratio of treated with 100 μmol/L DADS at 24 h, but they were both higher than the inhibition ratio of treated with 10 μmol/L SB203580 and 10 μmol/L Z-DEVD-FMK respectively (9.73% and 6.77%).

### Flow-cytometric analysis of apoptosis

**Figure 2:**
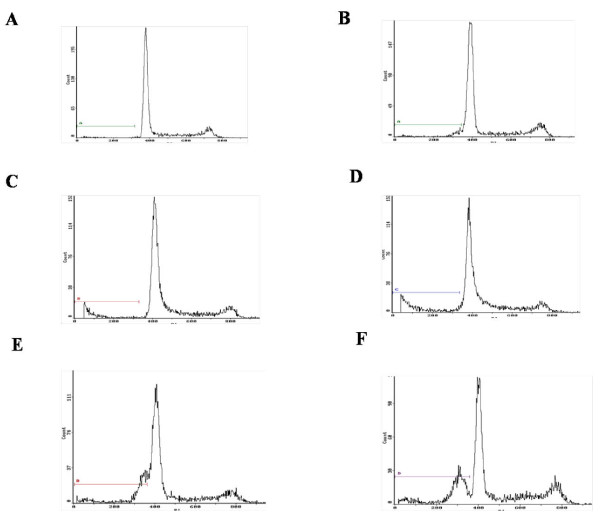
**Effects of each group on apoptosis in in human HepG2 cells**. A. Control (untreated), B. Z-DEVD-FMK (10 μmol/L), C. SB203580 (10 μmol/L), D. treated with DADS (100 μmol/L) after being treated with SB203580 (10 μmol/L) for 30 min, E. treated with DADS (100 μmol/L) after being treated with Z-DEVD-FMK (10 μmol/L) for 30 min, F. DADS (100 umol/L). Results are representative of three replicates (P < 0.01).

**Figure 3:**
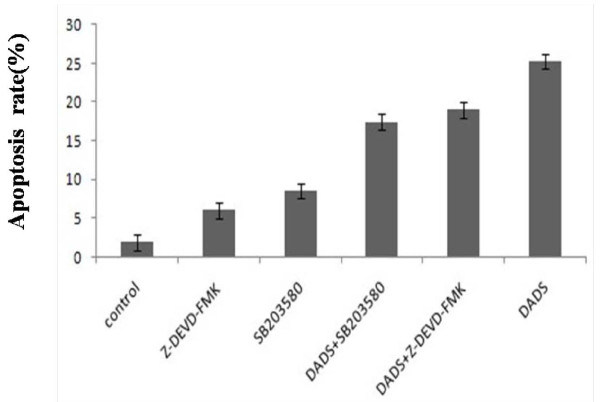
**Results of the flow cytometry analysis**. Data are expressed as mean ± S.D and evaluated by one-way analysis of variance (ANOVA). The results are representative of three independent experiment.

The results of flow cytometry analysis showed, the rate of SB203580-DADS group and SB203580-Z-DEVD-FMK group was 18.98% and 17.45% respectively, 1.86% of control group, 8.50% when treated with SB203580 (10 μmol/L), 6.02% when treated with Z-DEVD-FMK (10 μmol/L), and 25.23% when treated with DADS (Figure 2). These results suggested that inhibitors of P38MAPK and caspase-3 both had obvious effect of inhibiting apoptosis (Figure 3).

### Western-blot analysis

**Figure 4:**
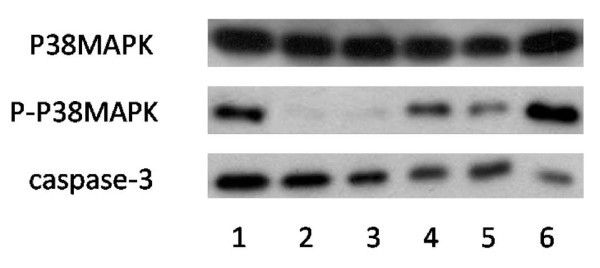
**Effects of each group on the protein expressions by Western blot**. Lane 1: control (untreated), lane 2: treated with DADS (100 μmol/L) after being treated with SB203580 (10 μmol/L) for 30 min, lane 3: SB203580 (10 μmol/L), lane 4: Z-DEVD-FMK (10 μmol/L), lane 5: treated with DADS (100 μmol/L) after being treated with Z-DEVD-FMK (10 μmol/L) for 30 min, lane6: DADS (100 μmol/L). The results are representative of three independent experiment.

After various treatment for 24 h, the zymogen bands of caspase-3 treated with DADS (100 μmol/L) became thinner significantly compared with the control gtoup, proving that DADS could advance the activity of caspase-3; after treated with SB203580 (10 μmol/L) and Z-DEVD-FMK (10 μmol/L) respectively, the zymogen bands of caspase-3 became thicker significantly compared with treated with DADS (100 μmol/L), but compared with the DADS (100 μmol/L) group that 30 minutes ahead of schedule by adding inhibitor, the band is only slightly thinner (Figure 4).

Similarly, SB203580 (10 μmol/L) and Z-DEVD-FMK (10 μmol/L) had inhibition effect on the P-p38 MAPK, When SB203580(or Z-DEVD-FMK) was added to HepG2 hepatoma cells for 30 min before DADS treatment or only added SB203580 (or Z-DEVD-FMK) to HepG2 hepatoma cells, P-p38 MAPK was markedly decreased, but DADS induced activations of P-p38 MAPK, compared to DADS-treatment alone or no treatment (Figure 4).

**Figure 5:**
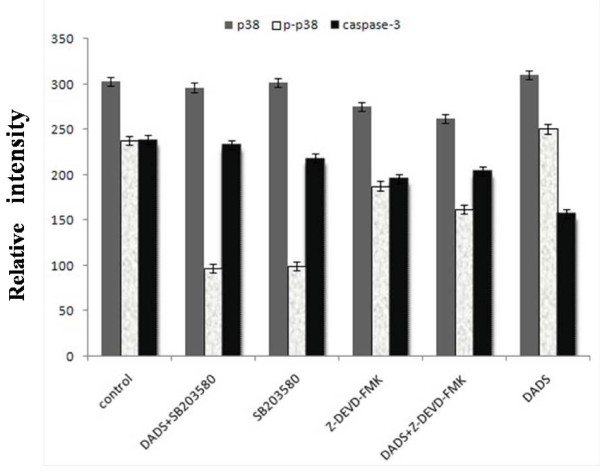
**Results of protein expression**. Data are expressed as mean ± S.D and evaluated by one-way analysis of variance (ANOVA). Results are representative of three replicates (P < 0.01).

These results confirm that SB203580 and Z-DEVD-FMK could inhibit the activity of P-p38 MAPK and caspase-3. But the inhibition of SB203580 was stronger than Z-DEVD-FMK, comparatively (Figure 5).

## Discussion

Apoptosis is a very complex process with the complexity and diversity, different cells in different stress have different signal transduction pathways. Extracellular signals how pass to cells and cause cells to the corresponding reaction is very important to the occurrence of apoptosis in the process of cell apoptosis.

The MAPK (mitogen-activated protein kinase) system is a cluster of intracellular serine/threonine protein kinases, playing an important role in a variety of signal transduction pathways of the mammalian cells. In recent years, many research report that apoptosis signal transduction and activation of caspase have a closely relationship, and have found 16 members of caspase family in mammalian cells [14-16]. All the Caspase exit in the form of inactive zymogen, can lead to caspase cascade reaction after be activitied, and eventually induce apoptosis. Undynamic caspase-3 will trigger apoptosis when it is activitied, and play a very important role when cells started apoptosis as the central effector of apoptosis [17-20].

Our previous work has demonstrated that DADS transiently activates both p38MAPK and p42/44MAPK while it induces apoptosis in a time and dose dependent manner in human HepG2 hepatoma cells[13]. The present study focuses on the role of p38MAPK and caspase-3 in cell apoptosis and DADS-induced apoptosis. To test the relation of p38MAPK and caspase-3 in the apoptosis process of human HepG2 cells induced by DADS, we used the inhibitors of p38MAPK (SB203580) and caspase-3 (Z-DEVD-FMK), the methods of MTT, flow cytometry analysis and western blot, The results presented in this study established a potential role for inhibitors of p38MAPK and caspase-3 in DADS-induced apoptosis. First, inhibitor (SB203580 or Z-DEVD-FMK) have the effect of inhibitory activity on p38MAPK and caspase-3. Second, a combination treatment with both DADS and inhibitor (SB203580 or Z-DEVD-FMK) decreases the inhibitory and apoptotic activity of HepG2 cells increased compared with DADS-treated (Figure 1, Figure 3, Figure 4 and Figure 5). The combined effect suggests a co-chemocytotoxic value in human HepG2 cells. In conclusion, our results show that p38MAPK and caspase-3 are involved in the process of DADS-induced apoptosis in human HepG2 cells and interact with each other.

At present, there have made some progress on the effect of MAPK signaling pathway in cellular apoptosis, but need in-depth study to fully reveal its mechanisms of action. Our results show that p38MAPK and caspase-3 are involved in the process of DADS-induced apoptosis in human HepG2 cells, enhance DADS-induced apoptosis and interact with each other, but its mechanism remains to be further discussed [21-24]. Further study the relationship of MAPK signal transduction pathway and caspase in the cellular apoptosis process, will have important significance for studying anti-tumor mechanisms of DADS and designing new drugs.

## Competing interests

The authors declare that they have no competing interests.

## Authors' contributions

FR, MX and CJ designed the experiments. CJ carried out most of experiments and drafted the manuscript. HM carried out partial experiments. All authors read and approved the final manuscript.
